# Systems analysis of miRNA biomarkers to inform drug safety

**DOI:** 10.1007/s00204-021-03150-9

**Published:** 2021-09-12

**Authors:** Amy L. Schofield, Joseph P. Brown, Jack Brown, Ania Wilczynska, Catherine Bell, Warren E. Glaab, Matthias Hackl, Lawrence Howell, Stephen Lee, James W. Dear, Mika Remes, Paul Reeves, Eunice Zhang, Jens Allmer, Alan Norris, Francesco Falciani, Louise Y. Takeshita, Shiva Seyed Forootan, Robert Sutton, B. Kevin Park, Chris Goldring

**Affiliations:** 1 MRC Centre for Drug Safety Science, Department of Pharmacology and Therapeutics, University of Liverpool, Sherrington Buildings, Ashton Street, Liverpool, L69 3GE UK; 2 bit.bio, Babraham Research Campus, The Dorothy Hodgkin Building, Cambridge, CB22 3FH UK; 3 CVRM Safety, Clinical Pharmacology and Safety Sciences, R&D, AstraZeneca, Gothenburg, Sweden; 4 Merck & Co., Inc, 770 Sumneytown Pike, West Point, PA 19486 USA; 5 TAmiRNA GmbH, Leberstrasse 20, 1110 Vienna, Austria; 6 GlaxoSmithKline (GSK), Stevenage, Greater Cambridge Area, UK; 7 ABHI, 1 Duchess St, 4th Floor, Suite 2, London, W1W 6AN UK; 8 Centre for Cardiovascular Science, The Queen’s Medical Research Institute, University of Edinburgh, 47 Little France Crescent, Edinburgh, EH16 4TJ UK; 9 Genomics EMEA, QIAGEN Aarhus, Prismet, Silkeborgvej 2, 8000 Aarhus C, Denmark; 10 Arcis Biotechnology Limited, Suite S07, Techspace One, Sci-tech Daresbury, Keckwick Lane, Daresbury, Warrington, WA4 4AB UK; 11 Wolfson Centre for Personalised Medicine, Department of Pharmacology and Therapeutics, University of Liverpool, Crown Street, Liverpool, L69 3BX UK; 12 Applied Bioinformatics, Bioscience, Wageningen University and Research, Droevendaalsesteeg 4, 6708 PB Wageningen, The Netherlands; 13 Computational Biology Facility, MerseyBio, University of Liverpool, Crown Street, Liverpool, L69 7ZB UK; 14 Department of Molecular and Clinical Cancer Medicine, University of Liverpool, Biosciences Building, Crown Street, Liverpool, L69 7BE UK

**Keywords:** microRNA, Biomarker, Drug Safety, Systems Biology, Toxicology, DILI

## Abstract

microRNAs (miRNAs or miRs) are short non-coding RNA molecules which have been shown to be dysregulated and released into the extracellular milieu as a result of many drug and non-drug-induced pathologies in different organ systems. Consequently, circulating miRs have been proposed as useful biomarkers of many disease states, including drug-induced tissue injury. miRs have shown potential to support or even replace the existing traditional biomarkers of drug-induced toxicity in terms of sensitivity and specificity, and there is some evidence for their improved diagnostic and prognostic value. However, several pre-analytical and analytical challenges, mainly associated with assay standardization, require solutions before circulating miRs can be successfully translated into the clinic. This review will consider the value and potential for the use of circulating miRs in drug-safety assessment and describe a systems approach to the analysis of the miRNAome in the discovery setting, as well as highlighting standardization issues that at this stage prevent their clinical use as biomarkers. Highlighting these challenges will hopefully drive future research into finding appropriate solutions, and eventually circulating miRs may be translated to the clinic where their undoubted biomarker potential can be used to benefit patients in rapid, easy to use, point-of-care test systems.

## The problem of adverse drug reactions (ADRs) and limitations of current clinical toxicity markers

Adverse drug reactions (ADRs) represent a huge healthcare and societal burden, accounting for roughly 6.5% and 6.7% of hospitalizations in the US and UK, respectively (Lazarou et al. 1998; Pirmohamed et al. 2004). When considering pharmaceutical safety of a drug, toxicity and clinical pharmacology are both assessed, as is its potential impact on multiple organ systems. Clinical diagnosis of an ADR is challenging due to variable presentations, and biomarkers play an important role in aiding diagnosis by helping determine organ specificity whilst informing on duration of the toxic event and its severity. Biomarkers are also essential during pre-clinical development, in both in vivo and in vitro systems, helping to demonstrate monitorability and allowing confidence of clinical monitoring to ensure patient safety.

Drug-related toxicity can be highly variable, with different injured organs leading to different pathological phenotypes. Drug-induced *cardiotoxicity* is difficult to diagnose and predict (Marrone et al. 2015), with manifestations including hypertension and arrhythmia that can lead to heart failure. Non-invasive diagnostics including echocardiography (ECHO) and MUGA (multiple-gated acquisition scans) (Simoni and Brandão 2017) aid detection of chemotherapy-induced cardiotoxicity via identification of damage-associated reductions in left heart ventricle function (Zuppinger et al. 2007), but application is limited by issues including intra- and inter-variability (Cardinale and Sandri 2015). Blood based biomarkers such as expression of brain natriuretic peptide (BNP) and cardiac troponins (cTns) have shown promise for early diagnosis (O’Brien 2008; Ferri et al. 2013; Lenihan et al. 2016; Shah et al. 2017). Troponins are especially promising, with elevations linked to left ventricular dysfunction (Cardinale and Scherrer-Crosbie 2017), however potential issues regarding translation into humans (O’Brien 2008; Tonomura et al. 2012) and non-specific expression may mean they are insufficient for clinical use (Nishimura et al. 2015; Defilippi and Seliger 2018).

Similarly, clinical markers currently used to diagnose drug-induced *kidney injury* such as blood urea nitrogen (BUN), glomerular filtration and creatinine based measurements are poorly sensitive and lack specificity as they can be modulated by external factors including age and diet (Waikar et al. 2012; Lopez-Giacoman 2015; Pavkovic et al. 2016). Early diagnostics are essential in providing effective treatment, such as for acute kidney injury (AKI) (Pavkovic and Vaidya 2016). Novel biomarkers β2-microglobulin (B2M), clusterin and kidney injury molecule-1 (KIM-1) have shown promise by outperforming BUN as a biomarker in relevant in vivo models (Kohl et al. 2020; Vlasakova et al. 2020). Furthermore, urine based novel markers α-glutathione-S-transferase, albumin and cystatin C may offer prognosis on change of function or damage to glomerulus or proximal tubular nephron segments (Kim and Moon 2012; Charlton et al. 2014). However variability, as with cystatin C in relation to age and inflammation, may again limit application of these novel markers (Séronie-Vivien et al. 2008; Charlton et al. 2014). The need for rapid diagnosis following AKI has led to suggestions that KIM-1 and neutrophil gelatinase-associated lipocalin (NAGL) could act as more specific and sensitive indicators of injury (De Geus et al. 2011; Lim et al. 2013). KIM-1 is stable in urine and has been shown to relate to severity of damage (Huo et al. 2010; Liu et al. 2016), indicating better sensitivity compared to serum creatinine (Tekce et al. 2015; Griffin et al. 2019). Similarly, urinary NAGL has shown potential in diagnosis and prognosis of post-surgery AKI patients (Cho et al. 2014). Despite promise, a lack of specificity for AKI means KIM-1 and NAGL may be better suited to a biomarker panel (Medić et al. 2016), reflected by their inclusion in an FDA qualified panel of six urine creatinine-normalized biomarkers also containing clusterin and cystain C to monitor kidney toxicity during early phase clinical trials (Sandelius et al. 2020).

Diagnosis of drug-induced *liver injury*, which currently relies on general liver injury indicators, represents a major clinical challenge. Detection of intracellular hepatocyte enzymes alanine aminotransferase (ALT) and aspartate aminotransferase (AST) in serum can indicate release following necrosis related to hepatocellular injury. Increase of total bilirubin (TBL) and measurement of alkaline phosphatase (ALP) further help to determine overall liver function and cholestatic liver injury, respectively. Diagnosis of DILI incorporates measurements of these enzymes based on Hy’s Law, where if ALT is ≥ 3 × the upper limit of normal (ULN) and TBL is ≥ 2 × ULN and there is no other likely cause of enzyme elevations such as viral hepatitis then DILI can be assumed (Hornby et al. 2014; Kullak-Ublick et al. 2017). This diagnosis of exclusion is generally considered insufficient in a clinical setting but is necessary here as enzymes can also be elevated following liver damage that is non-drug induced (Teschke and Danan 2016).

In addition to this limited diagnosis of exclusion, several issues with the enzymatic biomarkers used means clinical DILI assessment can be difficult. A lack of specificity is a major issue. Whilst ALT isoform 1 (ALT1) is relatively liver-specific, ALT2 is present in skeletal muscle, as is AST which is also seen in the kidney and heart, whilst ALP is present in bone. As a result aminotransferases can rise following skeletal muscle injury (Nathwani et al. 2005; Pettersson et al. 2008), and isoform specific assays to mitigate this issue are not routine in most clinical laboratories (Church and Watkins 2019). This lack of enzyme specificity is coupled with poor injury sensitivity. Transient aminotransferase increases can occur with drugs that are not hepatotoxic, which can often delay approval of safe drugs (Church and Watkins 2019). Furthermore, baseline variations in serum concentration have been indicated in twin studies under control of genetic and environmental factors (Bathum et al. 2001; Rahmioglu et al. 2009). Overall current DILI biomarkers do not correlate well with histopathological staging of injury, lack prognostic capability and struggle to distinguish between liver toxicity mechanisms (Shi et al. 2010).

Despite the limitations of currently used clinical DILI biomarkers, several novel biomarkers have begun to be validated in research including cytokeratin-18 (CK18), glutamate dehydrogenase (GLDH), osteopontin (OPN), macrophage colony stimulating factor receptor (MCSFR) and miR-122 (Church and Watkins 2019). Whilst some possess favourable characteristics versus current markers, they provide little insight into mechanisms of liver injury, although miR panels have shown promise in distinguishing between drug-induced and non-drug-induced phenotypes of liver injury (Yamaura et al. 2012; Krauskopf et al. 2017).

The associated limitations of biomarkers for detecting drug-induced injury in the organs described above mean biomarker improvements are desired, as are biomarkers for *neurotoxicity*, *dermatological toxicity* and *activation of the immune system*. Marrone and colleagues (2015) reviewed comprehensively the role of miRs in toxicity across many organ systems and how toxicity can alter miRs in these organs (Marrone et al. 2015). Therefore, here we will focus on the challenges in miR analysis and the application of miRs in a drug-safety setting.

## The potential of miRNAs in safety assessment

### The biogenesis and function of miRs

**Fig. 1:**
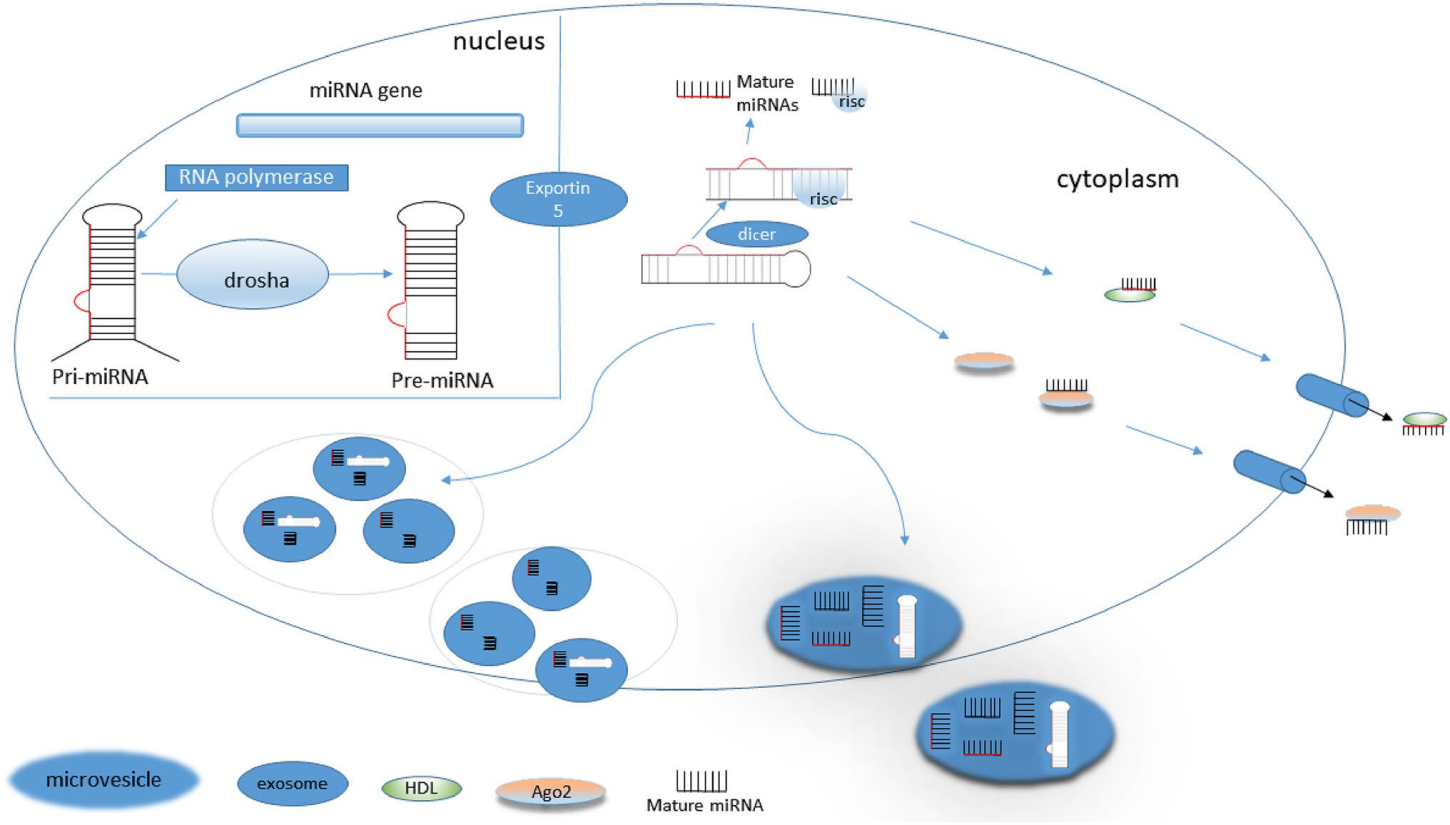
Basal miR biogenesis and secretion into the bloodstream. Long pri-miRNAs are initially transcribed from miRNA genes or can be co-transcribed with protein coding genes (Saçar Demirci et al. 2019) within the nucleus and translocated to the cytoplasm as immature pre-miRNAs by Exportin 5, where Dicer processes them into mature miRNAs (miRs) which can target mRNA for degradation or protein translation inhibition. Mature miRNAs can be associated with exosomes or coupled with Ago2 protein and released into the blood. Alternatively, they can be enveloped within microvesicles or attached to high density lipoproteins (HDL) and later released into the extracellular environment (Sohel 2016)

Mature microRNAs (miRs) are non-coding RNAs about 22 nucleotides long that take part in the RNA interference pathway, a mechanism that post-transcriptionally reduces gene expression. The biogenesis of miRs is seen in Fig. 1. miRs target mRNA by imperfectly base-paring to partially complementary 3’-UTR regions and promoting a reduction in their translation (Guo et al. 2010). This can be followed by mRNA deadenylation and de-capping, causing more rapid degradation of the target mRNA (Wu et al. 2006). The biogenesis and processing of miRs is tightly regulated, with miRs present in all metazoa and many sequences highly evolutionarily conserved (Lee et al. 2007). Almost all human mRNAs can be targeted by miRs—an example of this was seen in an evaluation of human Amyotrophic Lateral Sclerosis (ALS) gene regulatory pathways, where database analysis showed 99.15% of pathways had some form of miR-mediated regulation (Hamzeiy et al. 2018). miRs are among the fastest produced and longest-lived RNA species present in cells (Reichholf et al. 2019), and better understanding of miR function has indicated that miRs play a very important role in determining cell fate (Wilczynska and Bushell 2015). There is a strong bank of literature detailing the huge potential of miRs as biomarkers of toxic events. Here this review will detail the potential advantages of miRs as biomarkers, current evidence on their biomarker use, and the challenges that must be overcome prior to their introduction into drug-safety assessment.

### Potential advantages of miRs as biomarkers

There are several features of miRs which suggest potential for their use as biomarkers. One possible advantage of miRs over current biomarker options is cell specificity of certain miRs. Several miRs are only transcribed in one cell type, with database tools available to assess this specificity. One such tool is the RATEmiR database, an atlas cataloguing miR expression in major rat organs, which can compare tissue-enrichment and -specificity, as well as organ specificity. Here users can assess results from three bioinformatics pipelines, both alone and in combination (Bushel et al. 2018). miR specificity has been investigated in vivo, with identification of pancreas specific miRs in rat and canine models (Smith et al. 2016) and lung specific miRs in non-human primates (Yang et al. 2020). High conservation of miRs means results from in vivo studies are extremely relevant (Schraml et al. 2017), with translatable models like the beagle dog useful in helping to identify organ toxicity during early drug development (Koenig et al. 2016).

Another potentially beneficial aspect of miRs as biomarkers is that they can show a comparatively early response to toxicant exposure, even in events which are nontoxic, meaning miR alterations may occur under external stimuli prior to other more wholesale changes. These subtle exposure-related cellular events mean miRs may be useful in risk assessment (Marrone et al. 2014). Changes in miRs are apparent with cellular responses to stress (i.e. apoptosis/necrosis), following toxicity or with infection (Olejniczak et al. 2018). Both acute and chronic environmental exposure has led to miR alterations, showing them to be sensitive indicators of change (Vrijens et al. 2015). Alterations of miRs in such instances mean they are suitable candidates to act as markers of drug-induced tissue damage.

miRs can be released into the extracellular milieu through several mechanisms as shown in Fig. 1, and the nature of this release allows their detection in biofluids. Cellular miRs can be released passively due to apoptosis or necrosis, and later release can occur as miRs are trapped in apoptotic bodies (Howell et al. 2018). miRs released packaged in exosomes and associated/entrapped with vesicles or proteins have a degree of protection from extracellular RNases (Valadi et al. 2007; Harrill et al. 2016). As miRs are small in size they are often detected in blood as part of such complexes, with aforementioned protection thanks to macromolecules such as Ago2 protein (Arroyo et al. 2011) and high density lipoprotein (HDL) (Vickers et al. 2011). By forming such complexes miRs are fairly stable in biofluids such as whole blood and urine when properly stored, thus facilitating measurement from human plasma and serum (Mitchell et al. 2008; Mall et al. 2013). Complex formation such as with Ago2 may also have long-term storage benefits, as shown by circulating miRs being resistant against repetitive freeze–thaw cycle mediated degradation (Osaki et al. 2014), whilst miRs in formalin-fixed paraffin-embedded tissue are of suitable stability for analysis of archival material (Liu and Xu 2011; Boisen et al. 2015). Similarly, RT-qPCR analysis of serum miRs has shown no significant differences in results following miR exposure to pH extremes (Chen et al. 2008). This robust nature of miRs in biofluids is a key aspect in being suitable as a non-invasive biomarker.

Although general stability of miRs in biofluids support their use as biomarkers, it is important to note this is not a universal guarantee and there have been observations of free circulating miRs having differential stability between release states and between miRs themselves. As shown in Fig. 1 there are several potential states in which miRs can be released from the cell, this formation is important for miR stability as vesicle associated miRs have superior stability compared to non-vesicle associated miRs. Once present in serum miR species can also differ in stability, as during one 5-h incubation of the sera for example, where miR-122 was shown to degrade significantly whereas miR-16 did not (Köberle et al. 2013). Therefore, more detailed understanding of the stability of certain miRs in circulation may be necessary to maximize biomarker potential.

Sensitivity and specificity relating to drug-induced injury may be perhaps the biggest advantages of miRs as proposed biomarkers, as evident with studies involving miR-122 (Robles-Díaz et al. 2016), which has displayed superior biomarker performance in both aspects following human acetaminophen (APAP) toxicity compared to traditional enzymatic biomarkers. miR-122 has shown consistently to increase before ALT in serum (Thulin et al. 2014) and has been detected while liver enzymes were in normal range (Dear et al. 2014), whilst showing better sensitivity over aminotransferases in predicting APAP toxicity in patients presenting early to hospital (Vliegenthart et al. 2015). miR-122 has also shown high liver specificity, as highlighted in a study comparing miRs as potential liver and skeletal muscle drug-induced injury markers. Here, miR novel toxicity markers outperformed and added to sensitivity and specificity in detecting organ injury when compared to ALT in both cases, AST for liver and creatine kinase (CK) for skeletal muscle. This highlighted the capability of miR-122 to successfully diagnose DILI (Bailey et al. 2019).

The biological half-life of miRs is also a characteristic that may enhance its biomarker potential. Half-life of miR-122 in blood is estimated to be less than both ALT and AST, returning to baseline after 3–7 days, which may be indicative of progression and resolution of liver injury (Starkey Lewis et al. 2011). The nature and significance of miR half-life requires more research, such as by Matthews et al. (2020). Here, under inhibition of further hepatocyte miR production miR-122 was shown to have a shorter half-life than ALT despite a large endogenous release (Matthews et al. 2020).

### History of miRs as biomarkers of toxicity

The biochemical properties of miRs confer a strong advantage supporting their potential use as biomarkers. This is further supported by a number of relevant studies showing that miR detection can act as an appropriate marker for toxicity. Wang et al*.* first showed in 2009 that plasma and liver tissue of mice with acetaminophen-induced liver injury showed significant differences of miR-122 and -192 compared to control animals. These changes reflected histopathology and were detectable prior to ALT (Wang et al. 2009). Findings by Laterza et al. (2009) further highlighted the biomarker potential of miR-122. In rats treated with a muscle-specific toxicant aminotransferases increased, in contrast miR-122 showed no increase to this toxicant but did show a 6000-fold increase in plasma following treatment with hepatotoxicant trichlorobromomethane (Laterza et al. 2009). This pattern was later translated into humans, where a cohort of fifty-three APAP overdose patients had circulating miR-122 levels 100 times above that of controls (Starkey Lewis et al. 2011). miR-122 is the most abundant adult hepatic miR, accounting for approximately 70% of the total liver miRNAome (Bandiera et al. 2015; Howell et al. 2018), and has therefore become the best characterized potential miR liver biomarker, with a large research interest on its use as a circulating biomarker in response to drug-related hepatotoxicity (Zhang et al. 2010). Whilst there has been a strong focus on miR-122 as a marker of hepatotoxicity, research has also investigated miRs as toxicity biomarkers in other organs, with interest in circulating miRs as markers of toxicity from industry and amongst regulators. Several companies are currently at various stages of developing miR diagnostic panels, including for liver toxicity, brain disease and heart failure, with some currently available miR diagnostic panels including a panel for thyroid cancer (Bonneau et al. 2019).

### miRs beyond the liver

miRs have been researched as biomarkers of tissue damage for organs including the heart, brain, muscle and kidneys (Ji et al. 2009; Laterza et al. 2009; Vacchi-Suzzi et al. 2012; Akat et al. 2014). For cardiotoxicity miRs -1, -133, -34a and -208 have all been detected in serum following chronic administration of doxorubicin in mice and rats (Ji et al. 2009; Vacchi-Suzzi et al. 2012; Nishimura et al. 2015; Piegari et al. 2016). In terms of renal toxicity, miRs -21 and -155 can distinguish AKI patients when measured in urine, and have been shown as upregulated in the kidney following gentamicin exposure (Saikumar et al. 2012). Similarly, a panel of twenty-five miRs were decreased in the kidney and increased in the urine of rats treated with cisplatin (Kanki et al. 2014). Dysregulation in serum of CNS and hippocampus enriched miRs -9 and -384 following exposure to neurotoxin trimethyltin could suggest potential as biomarkers of CNS toxicity (Ogata et al. 2015), whilst significantly higher exosomal levels of miR-124 in acute ischaemic stroke patients means miR-124 could be a useful diagnostic and prognostic tool for ischaemic injury (Ji et al. 2016). Translatable plasma biomarkers of drug-induced pancreatic injury have been found in rat models, with miR-217-5p in particular showing high specificity and sensitivity, outperforming classical markers amylase and lipase (Erdos et al. 2020). Whilst single miR biomarker species are of significant interest, miR profiling studies have observed patterns of miR expression in a range of tissues, leading to research into measurement of miR panels as markers of injury (Ludwig et al. 2016).

There has been some criticism towards the characterization of widely-expressed abundant miRs as potential biomarkers, such as miR-21. miR-21 has been suggested as a marker for various diseases including coronary artery disease and hepatitis C, but it has been argued that a lack of specificity to any one disease means it cannot be considered a viable biomarker (Jenike and Halushka 2021). Whilst association with different disease states may limit application as a sole biomarker, assessment of miR expression in different tissues and even different cells remains useful to understand what variations in the circulation mean in the context of a disease. The changes of circulating miRs, even if not solely specific to a distinct disease state, can still help inform on indications and mechanisms of injury and damage and retain diagnostic potential perhaps in contributing to a detailed biomarker panel, which may have greater ability to differentiate between diseases.

As well as circulating miRs as markers for organ toxicity, some intracellular miRs are also being investigated as potential indicators of certain forms of intracellular perturbation, for instance potentially as biomarkers of mitochondrial toxicity (Baumgart et al. 2016).

Several examples of biofluid-detectable miRs whose levels are altered by chemical toxicants in different organ systems are given in Table 1.

**Table 1:** Biofluid-detectable miRs that are altered by toxicants in different organs. Adapted from (Schraml et al. 2017; Laterza et al. 2009; Wang et al. 2009; Saikumar et al. 2012; Haghikia et al. 2012; Yokoi and Nakajima 2013; Nassirpour et al. 2014, 2015; Ogata et al. 2015; Nishimura et al. 2015; Piegari et al. 2016; Raitoharju et al. 2016; Bergman et al. 2016; Koenig et al. 2016; Yan and Jiao 2016; Rouse et al. 2017; Bailey and Glaab 2018; Huang et al. 2018; Bailey et al. 2019; Erdos et al. 2020). The number of targets from miRTarBase to some of the miRs are shown in parentheses. It is of note that the numbers are very high. Arguably, unless the miRs with large target numbers occur abundantly themselves, effects may be difficult to measure. Thus, it would be beneficial to take target expression into account and monitor differential expression during marker development

miRs altered by toxicants in target organs that can be detected in biofluids					
Cardiotoxicity	Liver Toxicity	Kidney Toxicity	Neurotoxicity	Skeletal Muscle Toxicity	Pancreas Toxicity
miR-1-3p (900 +) miR-133a-3p (120 +) miR-208a/b-3p (60/60) miR-499a-5p (90) miR-34a-3p (90)	miR-122-5p (600) miR-192-5p (900) miR-103a-3p (400) miR-885-5p	miR-21-5p miR-155-5p miR-18a-5p miR-30a-c (900) miR-194 (200) miR-197 (1000) miR-200 miR-203 miR-320 Let-7d (400)	miR-9-3p miR-384-5p miR-922 miR-181c-5p miR-633 miR-150-5p miR-124a miR-124-3p miR-323	miR-133a miR-133b miR-1 miR-206	miR-216a-5p miR-216b-5p miR-217-5p miR-375-3p miR-148a

A summary of the putative main advantages and disadvantages of the use of miRs in general as biomarkers is shown in Table 2.

**Table 2:** A summary of the main advantages and disadvantages of using miRs as biomarkers of drug toxicity

miRs as biomarkers for use in drug-safety assessment	
Potential Advantages	Potential Disadvantages
Ubiquitous appearance in biofluids—serum, plasma, urine, saliva—enabling non-invasive sampling Tissue-specific expression patterns of certain miRs High sequence homology between animal models and humans facilitates translation of miR biomarkers – an important feature for pre-clinical development Enhanced stability in biofluids Availability on robust detection platforms such as RT-qPCR, next generation RNA sequencing, microarray platforms and biosensors enabling parallel quantification of multiple miRs Novel miR quantification methods being employed in research such as dynamic chemical labelling could facilitate point-of-care clinical detection Signatures unique to different aetiologies Can be measured in panels Prognostic and mechanistic value Knowledge of a wide range of expression levels of miRs as reflected in databases means miRs with low expression can be incorporated into panels	Measurement subject to sample quality and pre-analytical/analytical variability Lack of consensus regarding controls and standardization of assays Similar miR signatures resulting from many differing aetiologies Biological variability can be high and potentially influenced by smoking, diet and other environmental factors. Normal reference ranges therefore difficult to determine for some miRs No current clinical point-of-care assay Low levels of expression of many individual miRs

### Mechanistic and prognostic capability of miRs

miR-122 has some promising prognostic qualities in that it correlates more closely to histological grading of injury than ALT and seems to be predictive of whether a patient will recover or require transplant following injury (Ruoquan et al. 2014; Wang et al. 2016; Murray et al. 2017). However, rise of miR-122 with hepatitis C infection may affect this prognostic use, as it may reflect liver injury independent of aetiology. This issue may be solved by use of panels of multiple miRs, with miR panel profiles having potential to reflect the type of liver injury, such as differentiating between acute or chronic and hepatocellular or cholestatic phenotypes (Yamaura et al. 2012).

Glaab et al*.* (2018) demonstrated liver-specific (-122, -192) and muscle-specific (-1, -133a/b, -206) miRs outperformed, in terms of sensitivity and specificity, ALT and AST in monitoring the liver and AST and CK for monitoring skeletal muscle for drug-induced injury. The biomarkers were also able to sensitively monitor bile duct injury (necrosis and hyperplasia) seen with ANIT, methapyrilene and phalloidin. It was concluded that assessing a panel of miRs was an efficient and cost-effective investigative option (Glaab et al. 2018, unpublished data).

Measuring serum biomarkers to inform mechanistically about pathological states in organs is known as the concept of “liquid biopsy” (Lambrecht et al. 2018). In the case of DILI, Russo et al. (2017) showed miR changes were detected in the sera of acute DILI patients. Out of 3391 miRs and pre-miRNAs tested, eleven were significantly different between acute DILI patients and normal controls. miR-122 was the only miR deemed to have significant prognostic value, with the combination of miR-122 and albumin accurately identifying subjects who died within 6 months of DILI (Russo et al. 2017).

Another potential refinement for diagnosis of liver injuries when measuring miRs in the blood is their different fractionations under different aetiologies. miR-122 has been found to be predominantly in the exosome-rich fraction in alcoholic liver disease but mostly present in the protein-rich fraction during DILI (Bala et al. 2012). Another factor to help more detailed diagnosis could be analysis of structural miR variants known as isomiRs (isoforms of miRNA), as relative isomiR expression could further distinguish between disease states with several pathologies (Krauskopf et al. 2017). This has been evident in DILI where multiple miR-122 isomiRs were detected in patient serum but were at low concentration or not present in healthy counterparts. Importantly, PCR is shown to be inaccurate when analysing isomiRs, so alternative quantification such as dynamic chemical labelling (DCL) may be necessary (López-Longarela et al. 2020).

With regards to cardiotoxicity, miR-146a has shown dose-dependent upregulation in rats following exposure to chemotherapeutic doxorubicin, with overexpression of miR-146a in rat cardiac myocytes associated with reduced survival the cells (Horie et al. 2010). miRs have potential to distinguish between disease states within the heart, with dysregulation seen in acute myocardial infarction (Dimmeler and Zeiher 2010; Devaux et al. 2012), arrhythmia (Harling et al. 2017) and heart failure (HF), where reductions in circulating levels of let-7i, miRs -18a/b, -223, -301a, -652 and -423 have been associated with an increased risk of 180-day mortality (Ovchinnikova et al. 2016). Reduced levels of miR-145 have also been associated with the severity of coronary artery disease (Gao et al. 2015). This evidence indicates the prognostic potential of miRs in assessment of cardiotoxicity manifestations.

In terms of kidney injury, whilst KIM-1 is a promising urinary AKI biomarker (Shao et al. 2014; Pavkovic et al. 2016), it does not provide much insight into AKI mechanistically. Pavkovic et al. (2016) used target prediction to see targets of miRs associated with pathways perturbed across kidney pathologies. KIM-1 along with miRs -21, -200c, -423 were examined as candidate biomarkers of drug-induced AKI. The top pathway and associated pathological condition were found to be *MYC*-mediated apoptosis signalling and cell death and renal necrosis/cell death, indicating miR profiles can inform on mechanisms of damage in the kidney and intra-renal processes (Pavkovic et al. 2016).

## Roadblocks to using miRs in drug-safety assessment

### Pre-analytical challenges of using miRs in drug-safety assessment

#### Consideration of sample type

Whilst there is undoubted potential for miRs to act as useful biomarkers, there are several challenges to overcome before they can be employed as routinely as markers ALT and AST. Although non-invasive sampling is extremely useful (Howell et al. 2018), processing and extraction from these sources is crucial to miR measurements. Sample type can be diverse (Weber et al. 2010), ranging from plasma and serum to fresh or fixed tissue/tumours, purified by methods including immunoprecipitation or laser capture microdissection (Pritchard et al. 2012). Typically, isolation is performed via chemical extraction and purification using commercially available kits, and for low yield samples such as serum or plasma incorporation of a step determining the recovery of oligos spiked-in at extraction may be necessary. Once extracted, miR sample quality can be assessed, for instance utilizing spectrophotometer instruments in conjunction with a suitable normalization strategy (Becker et al. 2010). Such spectrophotometry approaches can be used to normalize total RNA content between samples, but this does not show a correlation with actual miR content (Wang et al. 2012).

Measuring miRs directly in serological samples is an area of promise. Bailey et al. (2019) utilized volume input as a normalization technique rather than isolated RNA or spike in calibrators. Here a consistent volume of plasma was used per-assessment, meaning data could be normalized against this volume and no further normalization was necessary. Results were then presented as fold-changes detected in treated animals relative to controls (Bailey et al. 2019).

Monitoring sample quality is extremely important as it can have a significant bearing on the validity of results. One such approach is quantification of isomiR content. These variants are characterized by changes in canonical miR sequence at the 3’ and/or 5’ end(s) (Dhanoa et al. 2019). miR degradation involves 3’ modifications which can affect miR steady-state (Neilsen et al. 2012). Therefore, levels of isomiRs in a sample may be indicative of the extent of 3’ modifications and sample degradation, as shown in patient serum in one study where miR-122 sample degradation produced increased levels of isomiRs. Here canonical miR-122 decreased over time with a concurrent increase of shorter isomiRs, with degradation enhanced under DILI (López-Longarela et al. 2020). There are several tools for isomiR analysis which can be incorporated to determine sample quality, including RNA-seq tool isomiR-SEA (Urgese et al. 2016), as well as CASMIR (Wu et al. 2018a) and miR-isomiRExp (Guo et al. 2016), which both focus on detecting relevant isomiR patterns.

Circulating miRs are often assessed using serum or plasma as the measurable biofluid, which requires centrifugation of whole blood (Bathum et al. 2001). Serum is cell-free liquid blood component following complete blood coagulation, whilst plasma is cell-free liquid blood component alongside an anticoagulant such as ETDA or citrate. This removal of cells is advised as cell contamination can impair miR quantification (Sohel 2016). Indeed, some investigations have attempted to discern which is preferable for miR measurement, although for cell type-specific miRs such as miR-122 a high correlation has been shown between serum and plasma profiles, whilst serum levels of miR-122 have shown to positively correlate with levels of major lipids (Willeit et al. 2017).

For serum a rich source of platelets means actual miR profiles can be biased towards that of platelets, meaning double-centrifuged plasma with a suitable anticoagulant may be prioritized over serum (Sunderland et al. 2017) as target miRs may be present in platelets. However platelet contamination has shown to also distort results in plasma, with one study showing it caused higher concentrations of miRs -15b, -16, and -24, although upon removal of cellular and subcellular components miRs became equivalent to that of serum (McDonald et al. 2011). In contrast inhibition of coagulation in plasma tubes has previously led to comparatively higher miR content detected in serum, with serum coagulation triggering miR release due to trafficking between cellular compartments and the extracellular environment. This again suggests properly centrifuged plasma may be preferable in comparison to serum for miR measurement (Wang et al. 2012), as shown by one assessment where although platelet contamination persisted despite tight control of lab practices it was minimized by following additional centrifugation steps (Cheng et al. 2013).

Platelet contamination is not the only preparatory condition that can affect plasma miR content. The degree of haemolysis is also crucial in determining the reliability of measurements of certain miRs. One such miR is miR-16, which is present in red blood cells with abundance and increase shown to be proportional to the degree of haemolysis, which therefore increases variability and reduces the capacity of red blood cell enriched miRs to act a references (Kirschner et al. 2011). Such lysis of red blood cells can be a critical cofounder of circulating miR analysis in both serum and plasma, and therefore should be monitored, with approaches including quantifying free haemoglobin by measuring absorbance at 414 nm or retrospectively measuring red blood cell enriched miRs such as miR-451 to detect erythrocyte rupture (Blondal et al. 2013).

**Fig. 2:**
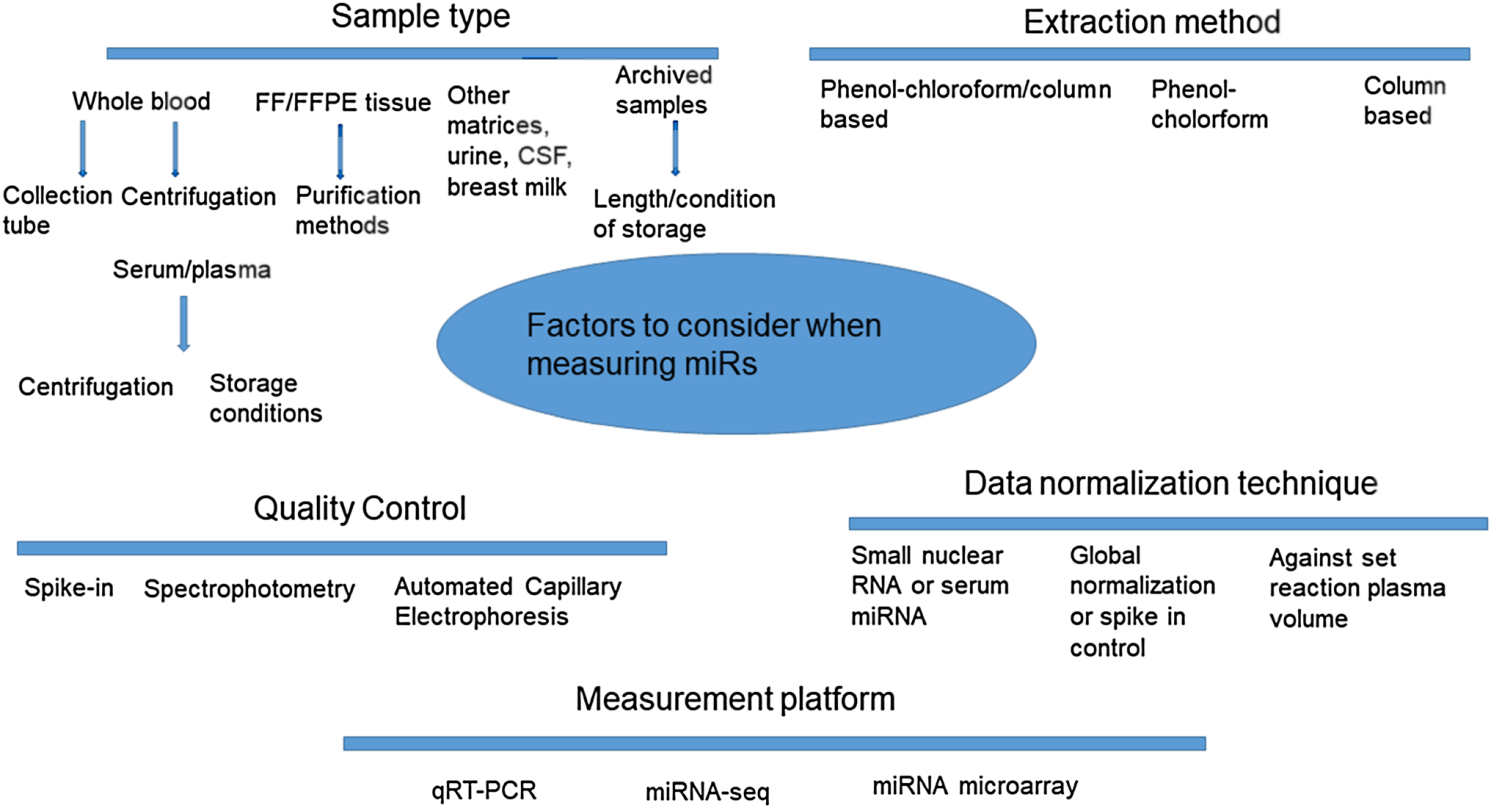
Factors to consider when measuring miRs that could potentially contribute to technical variability in miR bioanalysis. Both pre-analytical and analytical factors can contribute directly as well as indirectly to variation in the measurement of miRs across different platforms (Pritchard et al. 2012; Sohel 2016; Zhao et al. 2018; Bailey et al. 2019)

Lastly, it has been reported that using heparin tubes for processing plasma from whole blood should be avoided as this can result in reduced detection of miRs (Glinge et al. 2017) due to inhibition of PCR amplification (Willems et al. 1993). The numerous factors regarding sample type and processing and how they can influence miR measurement have led to a standardization protocol suggested by Glinge et al. (2017). Here, advice includes minimal sample shaking, separation of plasma/serum fractions and safe storage of aliquoted samples at − 80 °C (Glinge et al. 2017). Table 3 highlights a proposed exemplar protocol discussing preferable sample collection with the hope of minimizing the influence of the considerations discussed on miR measurement. Such quantitative variation is influenced by several factors, as shown in Fig. 2, and standardization of measurements is something to which all biomarkers must adhere to minimize variability and maximize reproducibility. The relative novelty of miRs as biomarkers means such standardization has yet to be agreed upon, but if a more consistent approach can be adopted as suggested in Table 3 then steps can be established and replicated across studies, helping sample type and collection issues to be minimized.

**Table 3:** To create a standardized way to process samples for the measurement of miRs, a universal protocol must be developed to address issues in variability caused by processing. This table shows a possible exemplar developed by the TransBioLine IMI consortium for processing plasma for miR analysis

A recent exemplar protocol that has been developed by the IMI TransBioLine consortium for prospective plasma sample collection for the purpose of miR analysis
1) Avoid haemolysis by following best practices
* Use good and consistent sample collection devices throughout a study (e.g. BD Vacutainer)
* Follow manufacturer’s instructions
* Avoid drawing blood from a hematoma
* Avoid foaming of the sample
* Make sure the venipuncture site is dry
* Avoid a probing, traumatic venipuncture
* Avoid prolonged tourniquet application or fist clenching
* Use correct size needle (~ 22 gauge)
* Fill vacuum tubes completely
2) EDTA anticoagulant. EDTA is most commonly used and available across labs. It is compatible with the protocols from other assay providers
3) Storage temperature between collection and centrifugation should be 4 °C. Our data suggest that cooled storage can reduce platelet activation and might improve stability of non-platelet miRs during longer storage times
4) Recommended storage times between blood collection and centrifugation/frozen storage was set to within 2 h
5) Double-centrifugation of plasma for complete removal of platelets. The first centrifugation step is performed at 2000×*g* (instead of 1000×*g*), to be compatible with plasma collection for protein biomarker analysis and hence facilitate the lab process and reduce errors
6) Storage and shipment of plasma in frozen state (− 80 °C and dry ice, respectively)

#### Pre-analytical standardization

As discussed, choosing sample type, for instance between serum and plasma, can have a significant effect on the results of miR measurements, as can both phlebotomy protocols and sample processing. Standardization is essential as reproducibility can be compromised by assay imprecision, especially during RNA extraction, meaning a reliable sampling procedure is vital. Whilst miRs are protected from RNases when they are released into the extracellular milieu, they can degrade quickly when spiked back into plasma, meaning certain sample types may require extraction methods that quickly inactivate endogenous RNases (Mitchell et al. 2008; Pritchard et al. 2012). miRs that are associated with vesicles, exosomes or Ago2 can also be altered depending on sample processing, subsequently influencing the measurement of some miRs (Arroyo et al. 2011), again highlighting the importance of correct sample processing.

Methods of extraction, as seen in Fig. 2, typically involve commercial phenol–chloroform or column based (or both combined) extraction kits. Different extraction methods have been compared in literature. In one comparison of five extraction methods, whilst all were suitable at extracting sample miRs, a high variability was seen between recovery of spike-ins, possibly indicating variability in RNA extraction efficiency (Brunet-Vega et al. 2015). It has also been reported when comparing methods that a combination of phenol–chloroform with a silica column based solid extraction method was preferable with respect to miR yield and integrity (Brown et al. 2018).

In the event of measuring miRs from archived samples then several sample and storage conditions must be considered to generate reliable results. Quality of the initial sample and age limit of samples may dictate whether the historical samples can be accurately investigated. If samples are prospectively collected in a quality study then the process should be described in the associated literature with details on time of sampling, blood tube used, if samples were on ice during processing and analysis as well as centrifugation speed, time and temperature. miRs have shown robustness at ultra-low temperature storage, for example one sample-set was stored without issue for seventeen years (Matias-Garcia et al. 2020), however details such as time from sampling to storage at − 20 or − 80 °C, time spent in freezer until analysis and number of freeze thaw cycles are all still important. Quality of historic samples could be further assessed by incorporating routine isomiR quantification using control samples, with increased isomiR presence correlating with miR degradation (López-Longarela et al. 2020).

RNA integrity is another factor which can impact the outcome of RT-qPCR analysis, and evaluating integrity is recommended as a routine step in pre-PCR miR analysis as total RNA integrity can interfere with techniques such as miR quantification, thus potentially compromising expression profiling of miRs (Becker et al. 2010). RNA integrity should therefore be monitored to allow consistent results, especially in archived samples.

For miR measurement to reach a confidence level where it can be routinely applied in the clinic pre-analytical variability as discussed here must be minimized by incorporation of more standardized, simplified approaches. The addition of a known concentration of exogenous synthetic miR before RNA extraction for instance represents a step to increase reproducibility and measurement confidence, meaning variations in RNA expression from results are more likely to be biologically meaningful and less likely to be due to experimental variability such as during RNA isolation or cDNA synthesis.

One example of researchers adopting more standardized and reliable approaches in miR measurement is by Glaab et al*.* (2018). Investigators evaluating the performance of liver and skeletal muscle-specific miRs versus traditional aminotransferases to detect DILI in rats recognized several challenges in isolating and measuring miRs from serum or plasma samples. The need for large plasma volume, limited miR endpoints, and normalization issues such as differences in plasma RNA levels due to toxicity, variability in total RNA isolation and potential need for a spike in control all impacted pre-analytical approaches. To overcome these difficulties a method was developed and optimized where a small 10 µl aliquot of plasma/serum was diluted in 100 µl water that was then applied directly into the reverse transcription reaction, without isolating the RNA beforehand. This addressed normalization and isolation artefacts and was used for all later miR analyses (Glaab et al*.* 2018, unpublished data). Such minimizing of pre-analytical variability may be essential for miRs reaching a reproducibility level suitable for the clinic.

### Analytical standardization

Pre-analytical considerations can have a major effect on result outputs from miR investigations, and so too can the analysis platform chosen for such miR profiling. For any biomarker to be clinically viable for drug-safety assessment it requires a reliable and robust detection platform. Current options for miR detection each have positive and negative aspects in terms of range, sensitivity and cost (Pritchard et al. 2012). A microarray approach is capable of identifying the expression of thousands of miRs in many species simultaneously (Liu et al. 2008), whilst RNAseq is highly accurate and can detect novel miRs, however it can display a lack of sensitivity for certain sample types (Kelly et al. 2013). Perhaps more appropriate to a drug-safety assessment setting is RT-qPCR, which can provide absolute quantification and (in-lieu of an easy-to-use point-of-care testing system) is less reliant on computational expertise.

Quantifiable metrics were used to compare the three analysis platforms to assess their sensitivity, specificity and reproducibility when measuring 196 different miRs as part of the miR quality control study (miRQC). Here, Mestdagh et al. (2014) concluded that approaches should be used in tandem such as RT-qPCR validation of screening experiments. qPCR platforms were shown to have greater sensitivity overall, especially when dealing with low-input RNA samples such as body fluids (Mestdagh et al. 2014). Whilst the approaches selected for determining miRs in biofluids are well established, certain technical aspects in the methods used require more universal standardization in order for measurements to become reliable in the eyes of regulators. Sufficient standardization and clinical data assessing a wide range of compounds and pathologies alongside traditional biomarkers will be vital in helping miR measurements becoming viable in routine assessment.

Normalization of results is important for any biological measurement to be reproducible and reliable. For miRs this is especially important, with RT-qPCR requiring a robust reference gene stable across all samples, as differences must be comparable to quantify measurements relevant to significant changes. Standardization is essential, as studies have described conflicting data when using different normalization strategies, with different methods leading to different outputs. This is evident with addition of exogenous oligonucleotides such as cel-miR-39, which correct for qPCR data related to processes such as RNA extraction but not for other factors to which it is not exposed. This represents an obstacle to miR profiling becoming common use in drug-safety assessment, and such factors must be kept in mind to select a reliable approach and thus generate reliable data (Faraldi et al. 2019).

A common normalization approach is versus an endogenous control gene which can correct for variables including differences in starting quantity. Ideally the endogenous control should be stable and extracted and quantified in the same fashion as the target miR (Das et al. 2016). Although PCR measurements commonly use endogenous controls such as beta-actin or GAPDH these are unsuitable for RNA analysis. This means selection often relies on previous studies, with a common choice being U6- (RNU6B), a small nuclear RNA molecule of the same class (Que et al. 2013; Wang et al. 2014). Despite regular use U6- has been shown to be unsuitable as a reference due to high variability between samples, in both healthy and patient groups (Benz et al. 2013; Xiang et al. 2014; Lamba et al. 2014; Masè et al. 2017). Analysis tools such as Normfinder, Genorm and Bestkeeper may be employed to select the most appropriate endogenous controls. Das et al. (2016) successfully used Normfinder to generate appropriate controls miR-25-3p and miR-93-5p for measurements from cancer cell lines (Das et al. 2016), whilst such tools allowed selection of optimal endogenous controls including let-7a and miR-103 for measuring exosomal serum samples in healthy individuals and hepatitis B/hepatocellular carcinoma patients (Occhipinti et al. 2016). Combining endogenous controls, such as let-7d/g/i, has also shown promise, with this option superior for serum miR measurement over U6- and RNU44/48 (Chen et al. 2013). There is no one common endogenous gene that can be used for all miR measurements, meaning selection of stably expressed miRs such as miR-152 or miR-23b (Lamba et al. 2014) or synthetic additions from organisms such as *C.elegans* may be more suitable normalizers.

The issue of normalization standardization has led to several studies looking at new approaches. One such approach is measurement of fold-change ratios of different miRs under pathology. Ratio measurements are used to classify DILI subtypes, with presentations determined by ratios of liver enzymes defined as the *R*-Value. López-Riera et al. (2020) quantified miR serum levels as fold-changes measured at admission and remission, and then incorporated fold-changes of individual miRs into ratios between different miRs. Differences in individual ratios of miR-122/miR-451a and miR-122/miR-16, respectively, enabled correct separation of most patients into hepatocellular and cholestatic DILI groups on account of greater miR-122 induction in hepatocellular DILI and preferential miR-451a/-16 repression in cholestatic DILI. Here miR ratio values showed excellent predictive performance (López-Riera et al. 2020). This approach is significant as if diagnosis can be made on relevant changes between two quantifiable miRs then there is less reliance on a housekeeping standard. Such approaches may be important in overcoming current normalization limitations.

In order for miR measurements to be reliably used in drug-safety assessments there needs to be some element of consistency in normalization approaches employed. This is true for any drug-safety related measurement as results must be reliable across all patients and groups to make suitable conclusions. In terms of miR quantification, endogenous controls are common and can be tailored for specific studies, but no endogenous miR can be detectable and stable across all disease states. Therefore more appropriate options with better standardization potential may be exogenous spike-ins or volume normalization as shown in Fig. 2. There needs to be a conscious approach in miR measurement research to develop and select a consistent standardized measurement strategy across studies. This could involve combination of some of the approaches discussed here, for instance incorporating isomiR quantification into RNAseq sample pipelines as a measure of sample quality. If a more universal approach can be adopted this will lead to more reliable and reproducible analyses, which will represent a significant step towards miR measurement becoming a viable drug-safety tool in the clinic.

### Inter- and intra-individual variability in basal miR expression

For miR measurements to be reliable indicators of injury, a good understanding of the presence and significance of variation in their basal levels is required. Intra-individual variation has been assessed by Wu et al. (2018b), where circulating miR levels in repeated samples were collected from fifty-one healthy volunteers over a 6–12-month period. 185 miRs were detected in at least 10% of plasma samples, 69 in 50% and 28 in 90%. The levels of 75 miRs revealed an intra-class coefficient (ICC) of > 0.5 when analysed in a subject at two time points 6 to 12 months apart, suggesting reasonable similarity, with a median ICC of 0.4 for the total 185 miRs. Notably, ICC was higher for miRs with higher plasma levels or higher detection rates (Wu et al. 2018b).

A study observing variability of DILI biomarkers by Church et al. (2019) included discussion of miR-122 which was shown to have a wide intra- and inter-subject variability across healthy volunteers (Church et al. 2019). Similarly, another study describing variability of miR-122 in serum found a higher-than-expected variability in healthy volunteers, with miR-122 producing a > 100-fold variation in two assessments whilst in comparison ALT variability was as low as fivefold. This large variability in healthy volunteers could have implications in the use of miR-122 as a DILI biomarker and therefore will need further investigation (Vogt et al. 2019). Indeed, this is an area requiring more research in general, it is important to find out if age, sex and race have a significant effect on the identity and quality of basal circulating miRs in large volunteer cohorts. Studies may therefore require collection of patient-baseline samples to perform intra-individual analysis. This could be possible in Phase I studies however it would not be feasible in a clinical scenario.

Although variation in healthy cohorts is an important consideration that requires further studies, context is important when relating its significance to the viability of miRs to act as circulating biomarkers, particularly miR-122. Observed miR-122 variations within a healthy group may be less significant in terms of biomarker performance due to the large dynamic range of miR-122, which is described as a key advantage in distinguishing false positives and false negatives of DILI. As shown by Starkey Lewis et al. (2011) the large signal of miR-122 under injury can remain significantly above the upper limit of the healthy cohort despite variability within that group, with minimal crossover between healthy and patient cohorts (Starkey Lewis et al. 2011). A similar trend was observed in a study looking at circulating miR-126 decrease as a biomarker for vascular dysfunction. Whilst large variation was seen in healthy and patient cohorts, miR-126 remained 88-fold lower in vasculitis patients than controls, suggesting its use as a potential vascular injury marker (Scullion et al. 2021).

### Beyond qPCR

There is a push to develop techniques in miR detection that do not require the specialized equipment, time and training as with the current options, with RT-qPCR for instance unsuitable for early diagnostics on a larger scale (Ouyang et al. 2019). Development of more suitable detection methods could mean a better chance of miR profiling being adopted in diagnostics.

With the aim of taking miR measurements to the bedside, whole blood measurements have been attempted to hasten their detection in the clinic. This is possible for miR-122 at least, as its high liver specificity means it is not expressed in cell types found in whole blood. This potential was highlighted in a study where finger venipuncture used to obtain capillary blood showed an 86-fold increase in miR-122 compared to healthy patients, with miR-122 levels comparable to that of plasma. This novel, near patient diagnostic test showed the potential for one blood drop to report DILI. Such point-of-care testing with easy access to transfer of miR-122 into testing could mean rapid DILI diagnosis and therefore quicker care (Vliegenthart et al. 2017). Another rapid and potentially cost-effective method for miR measurement is isothermal miR amplification. During amplification high quantities of H + can be generated, inducing significant changes in pH that can be monitored by pH sensitive indicators. Quantification is feasible as miR abundance is linked to the degree of indicator colour change, with this method comparable to RT-qPCR in successfully quantifying cancer cell miRs (Feng et al. 2017).

Another suggested alternative to RT-qPCR with reported significantly better sensitivity is droplet digital PCR (ddPCR), which has previous success in measuring plasma miRs as biomarkers for gastric cancer (Zhao et al. 2018; Ouyang et al. 2019). ddPCR has the potential to overcome current normalization issues, provide greater precision and be higher throughput, however when compared with qPCR for miR serum analysis results were largely concordant between the two methods (Campomenosi et al. 2016). The combination of a PCR step and a microarray identification step has also been implemented into a potentially portable prototype machine, requiring less sample preparation and showing enhanced sensitivity (Vaca 2014).

Development of an extraction-free, amplification-free miR-122 dynamic chemical labelling (DCL) detection assay also shows promise. The assay uses hybridization of miR-122 to an abasic peptide nucleic acid probe, which has a reactive amine replacing a specific nucleic acid, conjugated to superparamagnetic beads. This method was shown to identify patients at risk of DILI whilst displaying enhanced accuracy compared to PCR in terms of analysing miR-122 isomiRs. This is an advantage over current PCR assays which have variable efficiency across isomiR detection, suggesting a mix of isomiRs in a clinical sample may compromise accurate PCR quantification of miR-122 and other miR species. Addition of DCL beads to serum had the further advantage of stabilizing miR-122 signal for 14 days at room temperature, whereas signal degraded without beads (López-Longarela et al. 2020).

Another PCR-free technique for direct detection and quantification of miRs is Chemical Nucleic Acid Testing (Chem-NAT), which utilizes a labelled peptide nucleic acid capture probe with a reactive nucleobase that can base pair to the target miR, without requiring extraction of miRs from biological source. Researchers utilized this to formulate a Chem-NAT ELISA, which allowed accurate quantification of potential cancer biomarker miR-451a, whilst overcoming limitations of conventional miR analysis associated methods such as pre-extraction (Marín-Romero et al. 2018). The innovative novel approaches described here show how researchers are overcoming the challenges and limitations associated with current miR measurement techniques and represent promise in the effort to develop more clinically suitable miR diagnostic tools.

### The analysis of genome-wide circulating miR datasets

The potential of circulating miRs to function as early indicators of tissue damage encourages the systematic exploration of genome-wide analysis of the miRnome, currently comprising of over 2000 miRs (Kozomara et al. 2019). Ideally, similarly to other *omics* technologies, miR biomarkers are more valuable if they reflect a specific mechanism that may be relevant for the disease pathophysiology. Moreover, the complexity of miR regulatory networks, the tissue specificity and the timing of miR release suggests that considering combinations of multiple miR biomarkers is indispensable. Here we will look at some evidence in support of multi-miR marker signatures and discuss computational strategies that maximize the chance that such mechanistic biomarkers signatures are discovered from circulating miR genome-wide datasets.

A review on circulating miRs as cancer biomarkers suggested that single miR molecules could hardly meet the sensitivity and specificity criteria for candidate biomarkers (Wang et al. 2018). Regarding drug-induced liver injury, the extensively described and tissue specific biomarker candidate miR-122 still lacks specificity, as it is also altered in other liver pathologies. Combinations of multiple miRs, or even composite measures including other types of biomarkers, may have the potential of being more specific and being able to differentiate different pathologies (Johann Jr and Veenstra 2007; Zethelius et al. 2008; Martinelli et al. 2017). An independent validation study of previously postulated serum miR biomarkers for non-alcoholic fatty liver disease (NAFLD) confirmed the predictive value of miR-122 among other miRs, but found that 5 miRs (miR-192, -27b, -22, -197 and -30c) appeared specific for NAFLD when compared to DILI patients (López-Riera et al. 2018). The same study reported that models combining both clinical and miR variables showed improved predictivity. Another pilot study investigating serum miR biomarkers for diagnosis of cirrhosis and hepatocellular carcinoma (HCC) in hepatitis C patients found that a logistic regression model consisting of miR-122-5p and miR-409-3p was capable of distinguishing cirrhosis from mild disease, and that the prediction was improved by adding aminotransferase-to-platelet ratio (APRI) or Fibrosis 4 (FIB-4) clinical variables to the model (Weis et al. 2019). The study also showed that a panel consisting of miR-122-5p, miR-486-5p and miR-142-3p was capable of distinguishing HCC from cirrhosis while outperforming the only current biomarker alpha-fetoprotein (AFP).

**Fig. 3:**
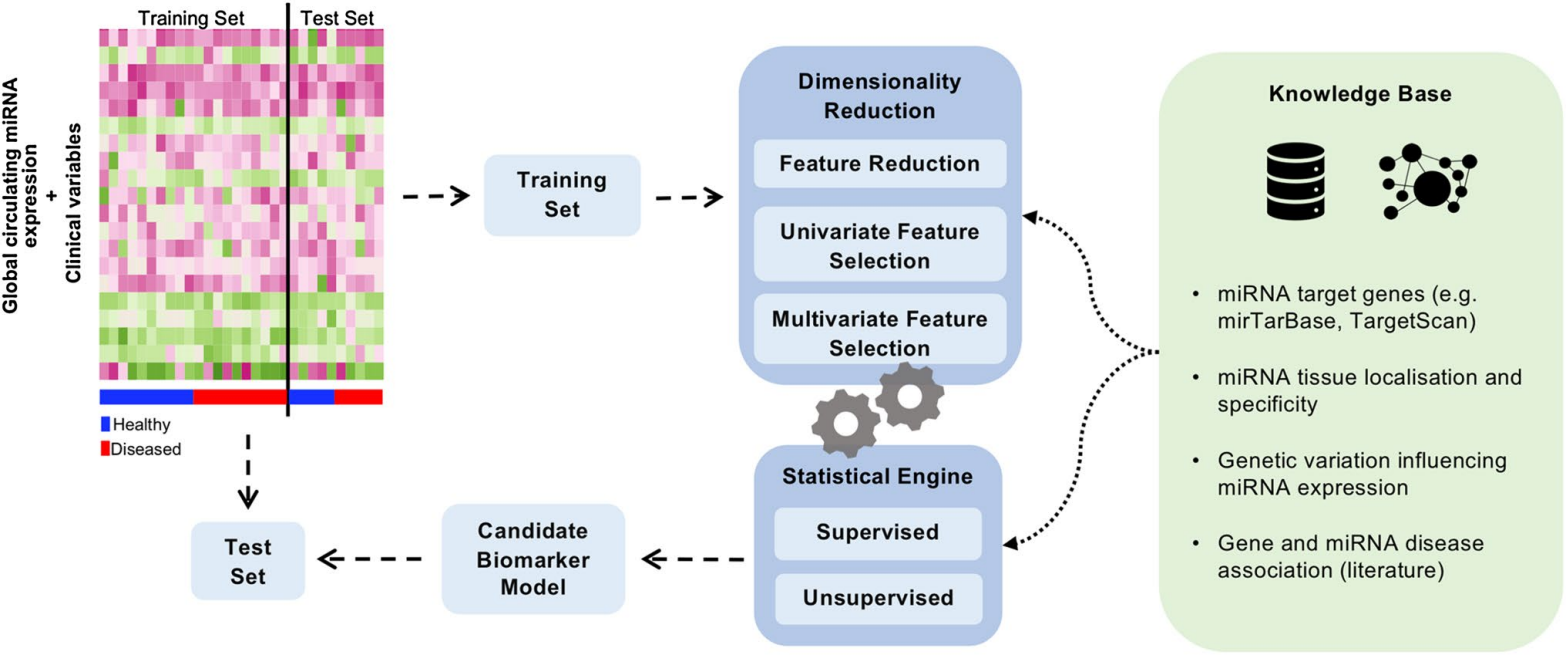
General pipeline for biomarker model development from global circulating miR datasets using knowledge-based approaches. Processed and normalized data is split into training and test sets, where the training set is used to build a model to predict outcome (healthy and diseased), while the test set assesses the ability of the model to correctly predict the same outcome in ‘unseen’ data. Prior biological knowledge can be incorporated in the algorithm for model development to increase the chances of finding an informative signature comprising of mechanistically-associated biomarkers

Altogether this supports the view that a sophisticated computational approach based on testing combination of miRs is of fundamental importance. Development of multi-biomarker models is typically based on multivariate statistical approaches, including machine learning approaches, and follows a general pipeline as detailed in Fig. 3. After data processing and normalization, generating predictive models involves splitting the data into training and test sets. The training set is used to build a model to predict outcome (e.g. categories of disease severity) while the test set assesses the ability of the model to correctly predict the same outcome in a dataset other than the one used to produce the model. An optimal biomarker model resulting from this process would be accurate in predicting outcome in both training and test sets. Due to the high dimensionality of these datasets, testing every possible combination of variables to identify the most predictive model is not a viable option, even with the computational power that is available. Therefore, the development of a predictive model must include a feature reduction or a feature selection step. Feature reduction involves combining the variables using a numerical transformation to obtain a smaller number of components that maximize the information. These components are then used as variables to develop the model. In contrast, feature selection involves selecting a subset of relevant variables to be included in the model. This step is not only important for reducing the computational time of the analysis, as it also decreases the chances of overfitting and allows the development of a biologically interpretable model. Several approaches can be taken to perform feature selection, such as the use of univariate procedures where each variable is tested independently, or multivariate variable selection procedures, designed to test combination of variables that maximize prediction. Multivariate variable selection procedures typically optimize variable subsets by progressive improvement of an initial random set by trial and error. During the process of optimization, biological knowledge can be used to develop a highly biologically relevant subset (Colaco et al. 2019).

Coupled to the dimensionality reduction component is the development of a prediction model. Generally, methods to develop a model are categorized as supervised or unsupervised learning, where supervised learning is applied for prediction of previously defined categories where data is labelled accordingly, whereas unsupervised learning clusters the data based on the naturally occurring patterns with no previously defined outcomes. In the context of biomarker development, mostly there is interest of distinguishing between pre-defined groups, where the application of supervised approaches is useful. Nevertheless, unsupervised approaches could provide insight in cases where there is uncertainty regarding classification categories (e.g. divergent classification systems for disease severity). For supervised approaches, the choice of the algorithm depends on the type of the pre-defined outcome. Categories (e.g. healthy vs diseased) require classification algorithms whereas continuous outcome variables require regression algorithms.

The methodology described above can be very effective, but since the procedure is unaware of the biological context of the marker, there is a chance of ending up with a highly predictive marker set lacking meaningful biological interpretation. Biomarkers containing functional relevance are more likely to be discovered if ‘knowledge’ is incorporated in the variable selection or in the process of model optimization. In the context of circulating miRs, prior knowledge such as known or predicted miR target genes (Singh 2017), tissue localization (Ludwig et al. 2016), miR gene promoters (De Rie et al. 2017), genetic variation influencing their expression (‘mirQTLs’) (Nikpay et al. 2019) and being part of a particular molecular pathway or gene ontology is information that can be used to drive the selection of biologically interpretable miR subsets. Several types of strategies can be used to incorporate these knowledge sources into model development, from simply selecting features matching specific criteria to generation of biological networks representing functional relationships. As an example, Vafaee et al. (2018) applied system-based approaches to identify plasma miR signatures predictive of prognosis of colorectal cancer patients. By integrating plasma miR profiles with a miR-mediated gene regulatory network containing annotations of relationships with genes linked to colorectal cancer, the study identifies a signature comprising of 11 plasma miRs predictive of patients’ survival outcome which also target functional pathways linked to colorectal cancer progression. Using the integrated dataset as input, the authors developed a bi-objective optimization workflow to search for sets of plasma miRs that could precisely predict patients’ survival outcome and, simultaneously, target colorectal cancer related pathways on the regulatory network (Vafaee et al. 2018). Since the amount of biological knowledge across different research fields is variable, and there is a lot yet to be discovered, alternative strategies could involve the application of algorithms that would increase the likelihood of selecting functionally relevant features while still allowing for the eventual selection of features based solely on their predictive power. This more balanced approach would allow for the selection of features with no known association to the outcome, which could be useful to biological contexts lacking extensive knowledge available and have the potential to reveal novel functional associations.

Thus, a plethora of strategies can be implemented to predict outcome from high-dimensional data. In the context of biomarker development, it is important that the decision-making process from predictive markers is understandable by researchers and interpretable by clinicians. This impacts the selection of methods to develop the model, favouring interpretable models (e.g. decision trees). This interpretability is being improved, for example use of a deep-learning based framework, where features can be discovered directly from datasets with excellent performance but requiring significantly lower computational complexity than other models that rely on engineered features (Cordero et al. 2020). Additionally, systems-based approaches that use prior biological knowledge can help in achieving this by guiding model development towards functionally relevant markers. One challenge presented in this area may be the analysis of multiple miRs in one test as a biomarker panel. Toxicity can be an acute presentation, and clinicians will need a quick turnaround in results. As already discussed, new assays may be needed and if a miR panel is of interest then multiple miRs will need to be optimized on the platform, further complicating a process that is already difficult for analysis of one miR of interest. This is something that should be kept in consideration when taking such approaches whilst looking at miR biomarker panels.

### Future considerations

Proof of the clinical utility of measuring miRs in drug-safety assessment is probably the major consideration in this field going forward. One of the issues of establishing miR measurements in a clinical setting is to increase the frequency of their use—part of the reason that this has not been the case is the lack of standardization in performance of the assays and reporting of the data. Pharmaceutical, clinical and regulatory organisations require reassurance of the reliability of biomarker measurements to utilize their full potential. Despite some favourable opinions by regulators, at present miRs, and notwithstanding some advantages over existing biomarkers, are not widely used in clinical decision-making. There is therefore an impetus for researchers to address fully the relative usefulness of these molecules as biomarkers. This includes the pull for Industry investigating the use of biomarkers to share exploratory data, thereby to increase the confidence in utilizing putative biomarkers in a clinical setting. To some extent this is now being done through US consortia as well as the Innovative Medicine Initiative biomarker pipeline programme, TransBioLine.

Standardization of miR measurements will be crucial if regulators are to accept miRs or indeed any other new biomarker class to be used alongside measurements employed currently. Clinicians, laboratories, and regulators need to collaborate to get to the stage where a point-of-care assay is agreed upon and adopted. As of now, this is unlikely to be through a qPCR format, as this is not time or cost -effective in a diagnostic environment. For the regulators of diagnostic assays used in a clinical setting, concerns centre around the fact that much of the evidence that miRs make effective biomarkers is based on the biomarker itself but not on the actual assay used for its measurement. Essentially support for miRs is attributed to their molecular characteristics, but questions remain about the application of the methods used for their detection in a routine clinical setting. Research is now needed to look at multiple panels of miRs and establish signatures that might be attributed to differing aetiologies. It will be important to determine if these signatures can also inform on progression and prognosis of drug-induced disease, by considering the dynamics of the miRs in question. In a very practical sense, miRs are generally well-conserved and this is important as it can obviate the need to spend time or money developing assays for biomarkers in different species. However, regulation hinges on the assay itself and its reliability—not just the exciting information that can be revealed by measuring the biomarkers themselves. Any clinically-used assay must be robust, inexpensive, relatively user-independent and have as short a turnaround as possible, with a ‘bedside’ test as the ultimate aim of biomarker research efforts. Whilst useful in a lab, the current approach of PCR-based measurement is simply too expensive for a bedside test, lacking cost effectiveness for larger-scale operation. This highlights how many of the challenges discussed here are reflective of the nature and regulation of biomarker use in drug-safety in general, and any novel marker must overcome such rigorous challenges to become suitable in a clinical setting.

Finally, considering the advantages of miRs as biomarkers, different signatures of miRs will need to be proven for their use in drug-safety assessment, i.e. that a signature is due to toxicity and not due to intra-individual or inter-individual variability, or another underlying condition or disease. Understanding these signatures in reference to drug-safety is going to require researchers to understand the meaning of these signatures in large healthy volunteer cohorts and different disease states. Implementing standardized measurement regimes may pave the way for miRs to achieve regulatory acceptance as biomarkers, and this challenge is being taken up in research as highlighted by the investigations into exciting new techniques as discussed here. Their potential as biomarkers (alone, in miR panels, or in combination with other molecules) has been established and remains a significant cause for optimism. Research is ongoing to help improve knowledge to facilitate miRs becoming viable clinical biomarkers. The strong interest in miRs as biomarkers of toxicity from regulators, industry and research can facilitate attempts to overcome the challenges currently restricting miR use in the clinic. If successful, this may unlock the clinical biomarker potential of circulating miRs in the future.
